# BRANCHED1: A Key Hub of Shoot Branching

**DOI:** 10.3389/fpls.2019.00076

**Published:** 2019-02-12

**Authors:** Ming Wang, Marie-Anne Le Moigne, Jessica Bertheloot, Laurent Crespel, Maria-Dolores Perez-Garcia, Laurent Ogé, Sabine Demotes-Mainard, Latifa Hamama, Jean-Michel Davière, Soulaiman Sakr

**Affiliations:** ^1^Institut de Recherche en Horticulture et Semences, Agrocampus-Ouest, INRA, SFR 4207 QUASAV, Université d’Angers, Beaucouzé, France; ^2^Institut de Biologie Moléculaire des Plantes, UPR2357, Université de Strasbourg, Strasbourg, France

**Keywords:** TCP transcription factors, hormones, nutrients, light, regulation, shoot branching

## Abstract

Shoot branching is a key process for plant growth and fitness. Newly produced axes result from axillary bud outgrowth, which is at least partly mediated through the regulation of *BRANCHED1* gene expression (BRC1/TB1/FC1). *BRC1* encodes a pivotal bud-outgrowth-inhibiting transcription factor belonging to the TCP family. As the regulation of *BRC1* expression is a hub for many shoot-branching-related mechanisms, it is influenced by endogenous (phytohormones and nutrients) and exogenous (light) inputs, which involve so-far only partly identified molecular networks. This review highlights the central role of BRC1 in shoot branching and its responsiveness to different stimuli, and emphasizes the different knowledge gaps that should be addressed in the near future.

## Introduction

Plants are sessile organisms that need to adjust their shape to suit the diversity of the changing environmental conditions in which they are growing. The regulation of shoot branching is a relevant strategy for plant survival and space occupancy, and involves an intricate regulatory network. Shoot branching depends on the status of bud dormancy, which is a temporary and reversible state (Shimizu and Mori, 1998). Shoot branching patterns, considered here as the distribution of branches along a parent stem, are generated during plant postembryonic development (Domagalska and Leyser, 2011). They depend on the ability of axillary vegetative buds located at the axil of each leaf to remain inactive or to produce a new branch in response to variable stimuli (Shinohara et al., 2013; Rameau et al., 2015; Wang and Jiao, 2018).

Shoot branching is an important feature of plant architecture that determines the interface between the plant and the surrounding environment. Shoot branching contributes to essential processes such as the establishment of leaf area and distribution that determine light interception and photosynthesis, which in turn influence the number of flowers and fruits, fruit filling and yield (Jiang and Egli, 1993; Richards, 2000). Branching also influences the plant competitiveness against weeds or the propagation of pests (Lemerle et al., 1996; Zhao et al., 2006; Simon et al., 2011). In ornamental plants, branching also determines plant visual quality, which drives consumers’ preferences (Ta et al., 1987; Boumaza et al., 2009, 2010; Garbez et al., 2015).

Extensive studies have been undertaken for several decades to find out the mechanisms involved in branching. The currently accepted idea supports that endogenous, developmental, and environmental inputs converge into bud-located integrators, which are at the head of a network of mechanisms governing the ability of buds to grow out. Among these inputs, hormones, sugar, nitrogen, light, and water play a determining role in shoot branching regulation (McSteen, 2009; González-Grandío et al., 2013; Niwa et al., 2013; Li-Marchetti et al., 2015; Rameau et al., 2015; Teichmann and Muhr, 2015; Corot et al., 2017; Le Moigne et al., 2018). Those factors may influence shoot branching via various physiological and molecular mechanisms, targeting different branching-related genes and acting synergistically or antagonistically. *BRC1* (*BRANCHED 1*) is well known to act locally in buds and is considered to be an important hub of different signals controlling the ability of a bud to grow out in many species (Aguilar-Martínez et al., 2007; Dun et al., 2009; Leyser, 2009; Beveridge and Kyozuka, 2010; Rameau et al., 2015). *Arabidopsis thaliana* harbors two *BRANCHED* genes, namely *BRANCHED 1* (*BRC1*) and *BRANCHED 2* (*BRC2*); they encode TCP transcription factors closely related to *TEOSINTE BRANCHED1* (*TB1*) in maize and *FINE CULM 1* (*FC1*) in rice. In addition, they are conserved in many species of the plant kingdom (Table 1). The corresponding mutants show an altered branching phenotype as compared to the wild type (Aguilar-Martínez et al., 2007; González-Grandío et al., 2013). This review addresses the molecular identity of *BRC1*, its involvement in shoot branching, and its regulation in response to endogenous inputs (hormones and nutrients) and exogenous cues (light). We also discuss how *BRC1* can mechanistically govern bud outgrowth, and raise a few questions about future investigations.

**Table 1:** The publication of *BRC1* homolog genes in different species.

Species		Name of the gene	Reference
Monocots	*Zea mays*	*TB1*	Doebley et al., 1997
	*Oryza sativa*	*Ostb1*/*FC1*	Takeda et al., 2003
	*Sorghum bicolor*	*SbTB1*	Kebrom et al., 2006
	*Hordeum vulgare*	*INTERMEDIUM-C*	Ramsay et al., 2011
	*Triticum aestivum*	*TB-D1*	Dixon et al., 2018
Eudicots	*Solanum tuberosum*	*StBRC1*	Nicolas et al., 2015
	*Pisum sativum*	*PsBRC1*	Braun et al., 2012
	*Dendranthema grandiflora*	*DgBRC1*	Chen et al., 2013
	*Arabidopsis thaliana*	*AtBRC1*	Aguilar-Martínez et al., 2007
	*Solanum lycopersicum*	*SlBRC1*	Martín-Trillo et al., 2011
	*Rosa hybrida*	*RhBRC1*	Barbier et al., 2015
	*Nicotiana tabacum*	*NtBRC1a; NtBRC1b; NtBRC1c; NtBRC1d*	Chen et al., 2016
	*Populus canescens*	*PcBRC1*	Muhr et al., 2018


## BRC1 Belongs to the TCP Transcription Factor Family

*AtBRC1* (also called *AtTCP18*) contains an open reading frame (ORF) made of *ca*.1,290-bp that encodes a protein with a TCP domain and an R domain. It belongs to the TCP gene family, an evolutionarily conserved family that first appeared in freshwater algae of the Charophyta family (Navaud et al., 2007). The TCP gene family was first described by Cubas et al. (1999) and is represented by four ‘founding members’: *TEOSINTE BRANCHED1* (*TB1*), *CYCLOIDEA* (*CYC*), *PROLIFERATING CELL NUCLEAR ANTIGEN FACTOR1* (*PCF1*), and *PCF2*, all identified on the basis of their functions in plant development or their DNA-binding capacities (for a review see Li, 2015; Danisman, 2016). In *Arabidopsis*, the TCP family comprises 24 genes encoding predicted proteins with a TCP domain (Cubas et al., 1999; Kosugi and Ohashi, 2002; Palatnik et al., 2003; Cubas, 2004) and categorized into two classes: class I (also known as PCF or TCP-P) is made up of 13 predicted proteins related to the PCF rice factors (Kosugi and Ohashi, 1997), and class II (also known as TCP-C) is made up of 11 predicted proteins related to the *Antirrhinum CYC* and *CIN* genes and to the *Zea mays TB1* gene (Luo et al., 1996; Doebley et al., 1997; Nath et al., 2003; Palatnik et al., 2003). All these transcription factors have the so-called TCP domain, a 59-amino-acid basic helix–loop–helix (bHLH), in common (Martín-Trillo and Cubas, 2010). Such a motif allows for DNA binding and protein–protein interactions in cells. The TCP domain is also necessary for nuclear localization (Kosugi and Ohashi, 1997; Cubas et al., 1999), and some TCP proteins can be targeted to the nucleus in heterologous systems (Suzuki et al., 2001; Qin et al., 2004).

Besides the TCP domain, a few class-II TCPs, including BRC1, display a functionally unknown arginine-rich motif, the R-domain, which is predicted to mediate protein interactions (Lupas et al., 1991; Cubas et al., 1999). The R domain may involve the phosphorylation process of BRC1 by a cAMP-dependent protein kinase (Dulhanty and Riordan, 1994; Martín-Trillo and Cubas, 2010). Additionally, most members of the *CYC/TB1* subclass, to which *BRC1* belongs, contain a conserved ECE (glutamic acid-cysteine-glutamic acid) motif that remains functionally uncharacterized and is located between their TCP and R domains (Howarth and Donoghue, 2006).

The TCP proteins of various species regulate many biological processes, including seed germination, plant branching, lateral organ development, floral asymmetry, gametophyte development, leaf senescence, circadian rhythms, and defense responses (for a review see Li, 2015; Danisman, 2016). These TCP-dependent regulations could occur directly through their binding to the promoter of target genes or indirectly *via* their interactions with plant hormones (Schommer et al., 2008; Guo et al., 2010; Danisman et al., 2012; Li and Zachgo, 2013; Nicolas and Cubas, 2016). In *Arabidopsis*, the *CYC/TB1* clade consists of *AtBRC1*, *AtBRC2* (also called *AtTCP12*) and *AtTCP1*, and is mainly involved in the development of axillary meristems, giving rise to either flowers or lateral shoots (Martín-Trillo and Cubas, 2010).

## BRC1 Is a Central Actor of Shoot Branching

The shoot axillary meristem produces a branch when the appropriate endogenous and exogenous inputs occur, so as to adapt plant architecture to environmental conditions. In monocots, *TB1* from *Z. mays* (Doebley et al., 1997) and homologs of *TB1* in *Oryza sativa* (OsTB1/FC1, Takeda et al., 2003) and *Sorghum bicolor* (SbTB1, Kebrom et al., 2006) promote bud arrest locally, without affecting the number of buds, and thus lead to reduced tillering. Consistently, *TB1* and *OsTB1* are mainly expressed in axillary bud meristems (Hubbard et al., 2002; Takeda et al., 2003), and their mutants *tb1* and *fc1* exhibit over-tillering phenotypes (Doebley et al., 1997; Wang et al., 1999; Takeda et al., 2003). The barley *TB1* ortholog, *INT-C*, has been shown to act mainly in the control of spike architecture, with a minor role in tillering (Ramsay et al., 2011). Moreover, modern maize displays less branching than the wild teosinte ancestor due to increased *TB1* expression (Studer et al., 2011; Zhou et al., 2011). However, the *int-c* loss-of-function mutant showed less tillers in barley, whose phenotype is opposite to the recessive *tb1* mutant in maize (Liller et al., 2015; Dong et al., 2019).

In dicots, genes closely related to *TB1* have been studied in a variety of species. In *Arabidopsis*, *AtBRC1* and *AtBRC2* both negatively regulate the branching process (Aguilar-Martínez et al., 2007; Poza-Carrión et al., 2007). However, *AtBRC1* seems to play a more pivotal role in axillary bud development than *AtBRC2*. The *AtBRC1* gene is predominantly expressed during the development of axillary buds (axillary meristems, bud leaf primordia and subtending vascular tissue). *AtBRC1* expression is inversely correlated with bud outgrowth and *brc1* mutant phenotypes are non-pleiotropic, while constitutive overexpression of *AtBRC1* reduces the growth of the whole plant (Aguilar-Martínez et al., 2007). Moreover, many *AtBRC1*-homologous genes have also been found to be involved in shoot branching suppression (Table 1). In addition, repressed buds in pea have been found to be as metabolically active as growing buds, so BRC1 growth repression may not involve metabolism (Stafstrom and Sussex, 1988). Recent data demonstrate that *AtBRC1* is not always necessary for the complete inhibition of all buds in *Arabidopsis* (Seale et al., 2017).

Genomic sequences of *Solanum* species, including potato and tomato, also contain the *BRC1*-like gene, where it occurs under two forms (Brewer, 2015). More interestingly, in *Solanum tuberosum*, the *BRANCHED1a (StBRC1a)* gene encompasses an alternative splice site leading to the generation of two BRC1a protein isoforms, BRC1a^Long^ and BRC1a^Short^, with distinct C-terminal regions (Martín-Trillo et al., 2011; Nicolas et al., 2015). The BRC1a^Long^ C-terminal region has a strong activation domain and moves to the nucleus, whereas the BRC1a^Short^ C-terminal region lacks an activation domain, which prevents the nuclear targeting of the protein (Nicolas et al., 2015). These different splice variants of *AtBRC1* have also been found in *Arabidopsis* (data not shown), but whether the mechanism mentioned above exists in *Arabidopsis* is still unknown. A central role of *BRC1* in shoot branching has also been revealed in pea (PsBRC1, Braun et al., 2012), *Chrysanthemum* (*DgBRC1*, Chen et al., 2013), and poplar (*PcBRC1*, Muhr et al., 2016 and 2018). In *Rosa* sp., Li-Marchetti et al. (2017) carried out a Quantitative Trait Loci (QTL) analysis of the plant architecture, using a segregating, recurrent blooming population called ‘The Fairy’ × ‘Old Blush’. They showed that the branching angle of order 2 long axes, the number of short axes (the type of axis that comprises one to four internodes), and stem elongation were correlated, with QTL located in the genomic region of *RhBRC1*, and assumed a pleiotropic role of *RhBRC1* in the establishment of the bushy shape of *Rosa* sp. Further work will be required to more accurately define the role of *BRC1* in the establishment of the plant complex architecture.

## BRC1 Is an Integrator of Diverse Hormonal Signaling Networks

Auxin, cytokinins (CK), and strigolactones (SL) are implicated in the hormonal regulation of *BRC1* expression. In this regulation network, auxin and SL act as inducers while CK act as repressors (Rameau et al., 2015; Teichmann and Muhr, 2015). According to Ferguson and Beveridge (2009), this kind of regulation could be involved in various metabolism pathways such as feedback regulation, long-distance hormone transport, and the interplay of plant hormone metabolism and signaling.

In apical dominance, the polar auxin transport (PAT) stream in the main stem, which is mediated by the PIN (PIN-FORMED) auxin-efflux facilitators located in xylem-associated cells (Petrášek and Friml, 2009), inhibits axillary bud outgrowth (Morris, 1977; Li and Bangerth, 1999; Zhang et al., 2007; Balla et al., 2011). Auxin cannot directly regulate *BRC1* expression because it is not transported from the stem to the buds in great enough amounts (Hall and Hillman, 1975). It is hypothesized that PAT prevents the establishment of auxin canalization from axillary buds to the stem, and that this might be necessary for the buds to grow out (Li and Bangerth, 1999; Domagalska and Leyser, 2011; Chabikwa et al., 2019). The characterization of the auxin-resistant *Arabidopsis* mutant *axr1* indicated that such an auxin effect occurred after axillary meristem initiation through the inhibition of bud outgrowth (Stirnberg et al., 1999).

**FIGURE 1:**
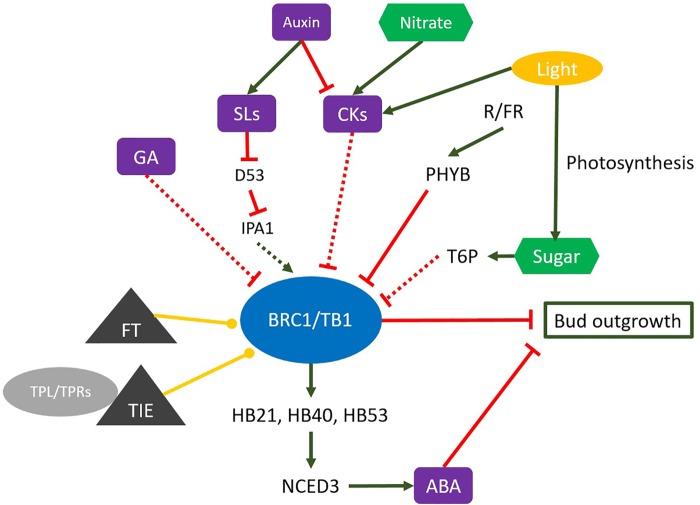
Many factors influence the expression of *BRC1*, including developmental, positional, genetic, hormonal, sugar signal and environmental factors. Auxin, cytokinin (CK), and strigolactone (SL) are implicated in the hormonal regulation of *BRC1* expression; auxin and SLs as promoters of *BRC1* and CKs as an inhibitor of *BRC1*. The red line, inhibition effect; the green arrow, stimulation effect; the yellow bullet-end lines, protein interaction; the violet element, plant hormones; the green element, plant nutrition; the green element, the yellow element, exogenous influence factor; the gray triangle, the proteins that interact with BRC1/TB1; D53, DWARF 53; HB21, HOMEOBOX PROTEIN 21; HB40, HOMEOBOX PROTEIN 40; HB53, HOMEOBOX PROTEIN 53; IPA1, IDEAL PLANT ARCHITECTURE1; NCED3, NINE-*CIS*-EPOXICAROTENOID DIOXIGENASE 3; PHYB, PHYTOCHROME B; T6P, trehalose-6 phosphate.

Auxin can indirectly promote *BRC1* expression in the bud (Aguilar-Martínez et al., 2007). Furthermore, auxin-mediated *BRC1* regulation through the control of two antagonistic factors, CK and SL, fine-tunes *BRC1* expression inside buds (Rameau et al., 2015). The role of CK, a collection of adenine-related compounds, in bud outgrowth was evidenced decades ago, when CK application to dormant buds was shown to promote bud outgrowth (Wickson and Thimann, 1958; Sachs and Thimann, 1967; Bangerth, 1994; Tanaka et al., 2006). In parallel, auxin indirectly inhibits bud outgrowth by decreasing systemic and local CK levels, which determines the CK supply to the buds (Miyawaki et al., 2004; Nordström et al., 2004; Tanaka et al., 2006; Müller and Leyser, 2011). CK can act to promote branching partly by promoting PIN3,4,7 cross-stem auxin transport between the bud and the adjoining stem, thereby potentially acting partly independently of *AtBRC1* repression directly in the bud (Waldie and Leyser, 2018). High CK levels in axillary buds lead to the activation of axillary buds through downregulation of *BRC1* expression (Braun et al., 2012), although *Psbrc1* (a pea *BRC1* mutant) remained sensitive to CK application. These findings might indicate that the branch-promoting hormone CK partly controls shoot branching by negatively regulating *BRC1* at the transcriptional level. In rice, transcript levels of *OsTB1*/*FC1* also decreased in a CK-dose-dependent manner (Minakuchi et al., 2010), and similar down-regulation of *DgBRC1* was reported in *Chrysanthemum* (Dierck et al., 2016). This CK-dependent *BRC1* regulation can be part of the light intensity-dependent bud outgrowth regulation in *Rosa* sp. (Roman et al., 2016; Corot et al., 2017). The *Arabidopsis altered meristem program1* (*amp1*) mutants are characterized by higher levels of CK, more bud outgrowth, more axillary meristems, and reduced *BRC1* expression (Helliwell et al., 2001). Although CK are a powerful repressor of *BRC1*/*TB1*/*FC1* expression, the molecular mechanisms driven by this CK-dependent regulation still remain an open question (Figure 1).

Strigolactones (SL), a group of carotenoids derived from terpenoid lactones (Lin et al., 2009; Alder et al., 2012), act as endogenous shoot branching inhibitors (Gomez-Roldan et al., 2008; Leyser, 2008; Wang et al., 2013). Direct application of GR24 – an SL analog – on buds inhibited outgrowth on intact and decapitated plants (Brewer et al., 2009), and auxin application elevated the transcription levels of SL biosynthesis genes (Sorefan et al., 2003; Foo et al., 2005; Johnson et al., 2006; Zou et al., 2006; Arite et al., 2007, 2009; Hayward et al., 2009). These findings support that auxin-mediated bud outgrowth inhibition involves the promotion of systemic and local SL synthesis in the stem and thereby of SL levels inside buds. Consistently, different SL mutants exhibited a highly branched phenotype in pea [*ramosus* (*rms*)], petunia [*decreased apical dominance* (*dad*)], and *Arabidopsis* [*more axillary growth* (*max*)] (Crawford et al., 2010). A role for BRC1 downstream of SL was first reported in *Arabidopsis* and pea, where *BRC1* expression was upregulated by SL, and shoot branching in the *brc1* mutant was insensitive to SL (Aguilar-Martínez et al., 2007; Dun et al., 2012; Revel et al., 2015). However, SL application did not change the transcriptional activation of *OsTB1/FC1* expression in rice (Minakuchi et al., 2010). Recent investigations showed that *DWARF 53* (*D53*)*/SUPPRESSOR OF MAX2 1-LIKE* genes (*SMXL6*, *7*, *8*) acted downstream of SL as repressors of SL-dependent *BRC1* upregulation and thereby promoted shoot branching (Jiang et al., 2013; Zhou et al., 2013; Kong et al., 2014; Wang et al., 2015). Mutants deficient in *D53*-like genes indeed displayed constitutive *BRC1* upregulation (Soundappan et al., 2015; Wang et al., 2015; Seale et al., 2017). Moreover, SL perception by D14 (α/β hydrolase) and the recruitment of the SCF complex resulted in the polyubiquitination and 26S-proteasome–mediated degradation of D53 (Kerr and Beveridge, 2017; Waters et al., 2017). D53 physically interacts with IPA1 (IDEAL PLANT ARCHITECTURE1), a repressor of shoot branching, and prevents it from upregulating *TB1* expression (Figure 1) (Song et al., 2017). IPA1, also named OsSPL14, is a member of the SQUAMOSA PROMOTER BINDING PROTEIN-LIKE (SPL) family of plant-specific transcription factors (Miura et al., 2010) that directly binds to the *TB1* promoter in rice and activates *TB1*transcriptional activity (Figure 1; Jiao et al., 2010; Lu et al., 2013). Further support for the relevance of the “*IPA-1*-related genes and *TB1*” module in shoot branching comes from a study in wheat, where TaD53 physically interacted with TaSPL3 and prevented TaSPL3 upregulation of *TaTB1* gene expression (Liu et al., 2017). Although the *Arabidopsis* homologs of *IPA1* have been identified as being *SPL9/15*, further work will be required to confirm whether this mechanism is involved in the SL-dependent regulation of *AtBRC1*.

Besides auxin, CK, and SL, gibberellin (GA) might also be involved in the regulation of *BRC1* expression, even if the mechanism is still unknown (Lantzouni et al., 2017). GAs (diterpenoid tetracyclin molecules) are plant hormones that regulate various developmental processes, including stem elongation, germination, dormancy, flowering, flower development, and leaf and fruit senescence (Hedden and Sponsel, 2015). In *Rosa* sp., GA biosynthesis strongly increases during bud outgrowth (Choubane et al., 2012). In the perennial woody plant *Jatropha curcas*, GA and CK synergistically promote lateral bud outgrowth, and both hormones negatively influence *BRC1* and *BRC2* expression (Ni et al., 2015). Simultaneously altered GA and SL levels positively influenced the expression of the *GA2 OXIDASE2* gene which encodes a GA-catabolic enzyme, and the expression of *BRC1* (Figure 1) (Lantzouni et al., 2017). Furthermore, GA is required for CK-mediated axillary bud outgrowth in *A. thaliana* (Jasinski et al., 2005; Lo et al., 2008).

## BRC1 Expression Is Regulated by Light

Shoot branching is negatively affected by low light intensity and low ratios of red/far red (R:FR) light in many species (Kebrom et al., 2006; Finlayson et al., 2010; Su et al., 2011; Revel et al., 2015). In this process, light acts both as a driver of photosynthesis for the supply of sugars to axillary buds and as a photomorphogenic signal (Su et al., 2011). The signaling role of light in plant branching was first unraveled by Kebrom et al. (2006). In 2006 and 2010, these authors showed that active PHYB suppressed the expression of the *SbTB1* gene in sorghum, leading to high plant branching, whereas environmental conditions that inactivate phyB (low R/FR ratio) increased *SbTB1* expression and in turn repressed bud outgrowth. Additional experiments carried out in *Arabidopsis* confirmed these findings: a low R/FR ratio favored *AtBRC1* upregulation through the PHYB pathway, which is required for shoot branching reduction (Figure 1; González-Grandío et al., 2013). This effect seems to be reversible, as evidenced by the rapid and local downregulation of *AtBRC1* after increasing the R/FR ratio (Holalu and Finlayson, 2017). Such a response may contribute to the rapid adaptation of plants to fluctuations in the R/FR light ratio.

Besides light quality, a slight decrease of the photosynthetic leaf area is associated with a stimulation of *TB1* expression in sorghum seedlings and consequently a lower propensity of tiller buds to grow out (Kebrom and Mullet, 2015). In addition, darkness-exposed *Rosa* sp. exhibited no bud outgrowth and higher levels of *RhBRC1* transcripts than plants placed under light (Roman et al., 2016). All these findings indicate that *BRC1* expression is very sensitive to both light intensity and quality. However, this regulation may involve distinct mechanisms (Kebrom et al., 2010).

## BRC1 Is Regulated by Nutrients

Sugars are well known to promote bud outgrowth in many species (Leduc et al., 2014; Rameau et al., 2015; Kebrom, 2017; Tarancón et al., 2017; Ferreira et al., 2018), and the relationship between sugars and bud outgrowth has been investigated for years (Maurel et al., 2004; Chao et al., 2007; Girault et al., 2010; Kebrom et al., 2010, 2012; Henry et al., 2011; Rabot et al., 2012; Mason et al., 2014; Barbier et al., 2015; Fichtner et al., 2017). Sugar effects are seemingly dependent on environmental conditions (Corot et al., 2017). Sugars not only serve as a carbon source for plant metabolism, but also as an important signaling entity that affects many developmental processes including *BRC1* gene expression (Price et al., 2004; Hellmann and Smeekens, 2014; Barbier et al., 2015; Sakr et al., 2018). In an interesting study, Mason et al. (2014) demonstrated that the initial signal responsible for the release of bud outgrowth after decapitation in pea was an increase in sugar availability rather than a decrease in apically supplied auxin, as traditionally thought. This is in line with the earlier proposal by Morris and collaborators (Morris et al., 2005), who assumed the existence of an auxin-independent “fast-decapitation signal” leading to bud outgrowth initiation after decapitation. Furthermore, Mason et al. (2014) also reported that the timing of the increase of the sugar flux inside buds and bud outgrowth tightly coincided with the downregulation of *BRC1* expression. In this process, sugar acts more likely as a signaling entity, because many non-metabolizable sugar analogs can trigger bud outgrowth (Rabot et al., 2012) and repress *BRC1* expression (Barbier et al., 2015). In addition, this effect of sugar on *BRC1* transcription could be mediated indirectly *via* sugar regulation of CK biosynthesis and SL signaling (Barbier et al., 2015) and/or directly (irrespective of hormonal action). Decapitation led to a rapid and sustained rise in trehalose-6 phosphate (T6P) levels in axillary buds and a decreased expression level of *BRC1*, which supports that T6P could partly mediate the sugar-dependent down-regulation of *BRC1* (Figure 1) (Fichtner et al., 2017). Further works are required to further unravel this molecular regulatory network. In the present state of knowledge, we cannot rule out that the transcriptional regulation of *BRC1* in response to sugars could involve many sugar-signaling pathways and also that *BRC1* expression is sensitive to the plant carbon status and/or energy levels (Martín-Fontecha et al., 2018).

Mineral nutrition influences tiller bud outgrowth in barley (Fletcher and Dale, 1974). In wheat, phosphorus deficiency directly altered the normal pattern of tiller emergence by reducing the rate of tiller emergence for each tiller (Rodríguez et al., 1999). Although several links exist between phosphate and the branching-related hormones (auxin, SL and CK), no direct effect of the phosphate status on *BRC1*/*TB1*/*FC1* gene expression is documented. Low-phosphate growth conditions enhance SL production in many species (Yoneyama et al., 2007; López-Ráez et al., 2008; Umehara et al., 2008; Domagalska and Leyser, 2011; Kohlen et al., 2011; Yamada et al., 2014). This situation leads to the repression of shoot branching (Umehara et al., 2008; Kohlen et al., 2011), but also to the stimulation of lateral root formation for soil foraging (Yoneyama et al., 2007; Ruyter-Spira et al., 2011). In contrast to SL, low levels of inorganic phosphate reduce CK production, which correlates with a reduced number of branches (Horgan and Wareing, 1980).

In herbaceous and woody plants, high levels of nitrogen fertilization (nitrate and/or ammonium) result in (i) a large number of outgrowing buds (Lortie and Aarssen, 1997; Médiène et al., 2002; Cline et al., 2006; Emarat-Pardaz et al., 2013; Pal et al., 2013; Furet et al., 2014), and (ii) improved secondary axis elongation (Thitithanakul, 2012; Thitithanakul et al., 2012). Luo et al. (2017) confirmed that nitrogen deficiency did not affect the initiation of tiller buds, but suppressed tiller bud outgrowth in *O. sativa*. In *Arabidopsis*, low nitrate delayed axillary bud activation, and this process involved an effect of the plant nitrogen status rather than a direct nitrate-signaling pathway (De Jong et al., 2014). Recent results demonstrated a relationship between nitrogen fertilization and *BRC1* expression in rice (Li et al., 2016). They showed that high ammonium nitrate intake in the root environment induced a reduction of apical dominance through overexpression of miRNA393 in the buds; miRNA393 inhibits the expression of the genes involved in auxin synthesis and signaling (*OsTIR1*, *OsAFB2*, and *OsIAA6*) as well as *OsTB1*. In *Arabidopsis*, the *brc1-2*/*brc2-1* double mutant exhibited a higher number of branches than the wild type, but low availability of nitrate reduced this effect (Seale et al., 2017). As root nitrate is widely known to induce CK biosynthesis and signaling events in the whole plant (Crawford, 1995; Sakakibara et al., 1998; Takei et al., 2001, 2002; Forde, 2002a,b), and CK repress *BRC1* expression, we cannot exclude that nitrate may affect *BRC1* expression through CK modulation. In rice, the supply of a CK analog (BAP) or ammonium nitrate regulated SL amounts in the stem and the bud within 3 h after treatment, but nothing has been reported regarding *BRC1* expression (Xu et al., 2015).

In Rosaceae as in many woody plants, nitrate is reduced and assimilated into amino acids directly in the roots; consequently, asparagine, arginine, aspartate, and glutamine are the main forms of nitrogen translocated to the buds via the xylem sap (Millard et al., 1998; Malaguti et al., 2001; Grassi et al., 2002; Guak et al., 2003; Le Moigne et al., 2018). In rose, asparagine is a major nitrogen form involved in bud outgrowth (Le Moigne et al., 2018); this is in accordance with previous data showing that application of asparagine on the soil of olive trees or on the leaves of poplar trees contributed to enhance bud outgrowth and secondary axis elongation (Proietti and Tombesi, 1996; Cline et al., 2006). In rice, a lack of cytosolic glutamine synthetase1;2 in the vascular tissues of axillary buds severely reduced their outgrowth (Funayama et al., 2013; Ohashi et al., 2015) independently of the SL level (Ohashi et al., 2015). In rose bush, sucrose, glucose, and fructose had to be associated to asparagine to allow for the buds to grow out *in vitro* (Le Moigne et al., 2018). This effect involved the upregulation of *IPT3* gene expression in the stem and in the vicinity of the bud (Le Moigne et al., 2018) and the downregulation of *BRC1* (Barbier et al., 2015). In addition to a nutritional role, asparagine might also be a signal representing the nitrogen status of the plant, so as to counteract *BRC1* expression through CK stimulation.

## A BRC1-Related Regulatory Mechanism

Many studies ascribe an inhibitory function of mitotic cell activity to BRC1 (Poza-Carrión et al., 2007; Kieffer et al., 2011). This is because early results of EMSA (Electrophoresis Mobility Shift Assay) revealed the capacity of the TCP domain to associate specifically with the promoter element of the rice proliferating cell nuclear antigen (PCNA) gene (Kosugi and Ohashi, 1997, 2002). These *cis*-regulatory modules are indispensable for the transcriptional activation of the PCNA gene in rice meristem tissues (Kosugi and Ohashi, 1997), which seems to be an ancient and prevalent role of TCP transcription factors (Ortiz-Ramírez et al., 2016).

BRC1-mediated branching is repressed by the regulation of abscisic acid (ABA) metabolism (Figure 1). ABA is a plant hormone that plays important roles in many phases of the plant life cycle (Seo and Koshiba, 2002; Hayes, 2018; Wang et al., 2018). Evidence for a role of ABA in regulating bud growth comes from the positive correlation between a reduction of ABA levels in buds and their release from dormancy (Cline, 1991). Moreover, the *Arabidopsis era1* (*ENHANCED RESPONSE TO ABA 1*) mutant exhibited high sensitivity to ABA and reduced branching (Pei et al., 1998). In *brc1 Arabidopsis* mutants, the ABA-signaling pathway showed a significantly reduced response as compared to the wild type. Additional data revealed that the expression levels of two ABA markers, *ABA-RESPONSE PROTEIN* (*ABR*) and *UDP-GLUCOSYL TRANSFERASE 74D1* (*UGT74D1*), were significantly upregulated in the wild type but not in *brc1* mutants treated with low R:FR light (González-Grandío et al., 2013). González-Grandío and Cubas (2014) support a model in which ABA acts rather downstream of BRC1, because *ABRE-BINDING FACTOR 3* (*ABF3*) and *ABA INSENSITIVE 5* (*ABI5*), two key regulators of the ABA response that contain TCP-binding sites in their promoters (Finkelstein and Lynch, 2000; Yoshida et al., 2010; González-Grandío et al., 2013; Nicolas and Cubas, 2016), are upregulated in axillary buds upon *BRC1* induction (González-Grandío and Cubas, 2014). They also indicated that BRC1 bound to and positively regulated three transcription factors: *HOMEOBOX PROTEIN 21* (*HB21*), *HOMEOBOX PROTEIN 40* (*HB40*), and *HOMEOBOX PROTEIN 53*(*HB53*). These three proteins, together with BRC1, enhanced *NINE-CIS-EPOXICAROTENOID DIOXIGENASE 3* (*NCED3*) expression, the main ABA-biosynthesis enzyme, leading in turn to ABA accumulation in buds (González-Grandío et al., 2017). This finding demonstrates a direct relationship between *BRC1* and ABA signaling, and places ABA downstream of *BRC1*. Consistently, *BRC1* expression was found to be insensitive to exogenous ABA application (Yao and Scott, 2015).

The TCP genes generally act by positively or negatively regulating the cell cycle (Sarvepalli and Nath, 2018). As a transcription factor, BRC1 could bind to the promoter region of various genes to regulate the branching process and participate to many regulatory mechanisms (González-Grandío et al., 2013). In maize, TB1 can directly activate the *tassels replace upper ears1* (*tru1*) gene that encodes an ankyrin-repeat-domain protein by binding to the promoter region of *tru1* (Dong et al., 2017). In *Arabidopsis*, bioinformatic analysis indicates that the promoter sequences of 1,950 genes expressed in the shoot bear the TCP-*cis* regulatory motif (5′-RRVMMMV-3′) and could be putatively regulated by *AtBRC1*. Based on Gene Ontology (GO) enrichment analysis, these putative target genes are thought to be mainly involved in metabolic processes, including amino acid metabolism [e.g., *ALANINE-2-OXOGLUTARATE AMINOTRANSFERASE 1* (*AOAT1*); *HYDROXYPYRUVATE REDUCTASE* (*ATHPR1*)] and sulfur (e.g., *sulfate transmembrane transporter* (*MOT2*), *sulfate transporter 1;2* (*SULTR1;2*)] (data not shown). We can therefore speculate that BRC1/TB1 might control bud outgrowth via various pathways, such as stimulating the ABA-signaling pathway and inhibiting cell division and cell metabolism.

## Conclusion and Perspectives

*BRC1*/*TB1*/*FC1* is an integrator gene involved in shoot branching, which fits well with the ability of *BRC1*/*TB1*/*FC1* expression to integrate many endogenous and exogenous inputs (Figure 1). However, the detailed mechanism whereby these stimuli regulate *BRC1* expression is still puzzling, and many mechanistic scenarios are plausible. Many questions are thus still open and include how CK and SL, the main two branching-related hormones, antagonistically regulate *BRC1* expression, and which molecular actors could be involved. Similar questions concern the sugar-mediated downregulation of BRC1, and the molecular mechanism behind the combined effect of nutrients and hormones on *BRC1* expression (Sakr et al., 2018). In addition, the regulation of gene expression includes many aspects, such as epigenetic regulation, transcriptional regulation, post-transcriptional regulation, translational and post-translational regulation. The relevance of these mechanisms in the regulation of *BRC1* expression deserves to be investigated in different biological contexts. Recent data showed that the protein interaction process also influences *BRC1* expression. For example, the florigen proteins FLOWERING LOCUS T (FT) and TWIN SISTER OF FT (TSF) influence axillary meristem development via their interaction with AtBRC1 (Niwa et al., 2013); TIE1 (TCP interactor containing EAR motif protein 1), a transcriptional repressor identified as involved in the control of leaf development, controls shoot branching by interacting with BRC1 (Yang et al., 2018). Additional protein partners may also interact with BRC1, including those related to the energy and nutrient statuses [Sucrose non-fermenting-related kinase (SnRK1)/Target of rapamycin (TOR kinase)] (Martín-Fontecha et al., 2018). Meanwhile, our knowledge about the molecular network governing the BRC1-dependent reduction of plant branching is still limited, and the only available data report that BRC1 action could be related to different biological functions such as cell proliferation, cell metabolism, hormone biosynthesis, ribosome biosynthesis, *etc*. All these findings indicate that further work is required to fully investigate the regulatory network behind the regulation and function of *BRC1* in shoot branching.

## Data Availability Statement

Publicly available datasets were analyzed in this study. This data can be found here: https://www.rosaceae.org.

## Author Contributions

All the authors listed have contributed significantly to this manuscript. MW, J-MD, and SS managed this review and contributed to different sections. M-DP-G and LO contributed to the TCP transcription factor section. JB, SD-M, LH, and LC contributed to both hormonal and environmental factors sections, and M-ALM contributed to the nutrient section.

## Conflict of Interest Statement

The authors declare that the research was conducted in the absence of any commercial or financial relationships that could be construed as a potential conflict of interest.
